# Predicting the canopy conductance to water vapor of grapevines using a biophysical model in a hot and arid climate

**DOI:** 10.3389/fpls.2024.1334215

**Published:** 2024-02-06

**Authors:** Ricardo Jorge Lopes Egipto, Arturo Aquino, José Manuel Andújar

**Affiliations:** ^1^ INIAV, I.P.—Instituto Nacional de Investigação Agrária e Veterinária, Pólo de Inovação de Dois Portos, Dois Portos, Portugal; ^2^ CITES, Centro de Investigación en Tecnología, Energía y Sostenibilidad, Universidad de Huelva, Huelva, Spain

**Keywords:** grapevine, canopy conductance modeling, water stress in grapevines, vineyard water management, sustainable irrigation

## Abstract

Canopy conductance is a crucial factor in modelling plant transpiration and is highly responsive to water stress. The objective of this study is to develop a straightforward method for estimating canopy conductance (g_c_) in grapevines. To predict g_c_, this study combines stomatal conductance to water vapor (g_sw_) measurements from grapevine leaves, scaled to represent the canopy size by the leaf area index (LAI), with atmospheric variables, such as net solar radiation (R_n_) and air vapor pressure deficit (VPD). The developed model was then validated by comparing its predictions with g_c_ values calculated using the inverse of the Penman Monteith equation. The proposed model demonstrates its effectiveness in estimating the g_c_, with the highest root-mean-squared-error (RMSE=1.45x10^−4^ *m.s^−1^*) being lower than the minimum g_c_ measured in the field (g_c obs_=0.0005 *m.s^−1^*). The results of this study reveal the significant influence of both VPD and g_sw_ on grapevine canopy conductance.

## 1 Introduction

Mediterranean viticulture faces numerous constraints, notably those associated with climate changes and less favorable meteorological conditions. The increasing frequency and severity of extreme climate events, such as warmer and drier climate conditions, and intense heat waves coupled with severe droughts, are exerting significant pressure on the available water resources. Projections for future climate scenarios anticipate a continued increase in the occurrence and magnitude of such extreme weather events. These changes are expected to have a profound impact on irrigation water supply systems, which are already under strain (IPCC, 2019). This scenario introduces new challenges to Mediterranean viticulture, where irrigation has emerged as a pivotal short-term adaptation measure. The effective implementation of grapevine irrigation requires a comprehensive understanding of the climate, soil, and water conditions specific to the vines. Additionally, accounting for their variability within the vineyard is essential, to manage the vine water stress while optimizing yield and berry composition (Chaves et al., 2016; Costa et al., 2016).

The Penman–Monteith method, describing evapotranspiration, considers soil and air humidity, mass transport and the energy required for the process (Monteith and Unsworth, 2013), and reflects the weather-related effects on crop water use (Allen et al., 1998; Pereira, 2004). However, this method has limitations related to soil and canopy conductance measurement (Paço et al., 2006; Picón-Toro et al., 2012; Rallo et al., 2021). Indeed, some of these limitations have been reported in the literature for sparsely planted, drip-irrigated crops, with significant exposed soil and a stringent stomatal control mechanism for water loss, such as grapevines (Shuttleworth and Wallace, 1985; Campos et al., 2010; Pereira et al., 2015; Schymanski and Or, 2017; Forster et al., 2022).

A simplified method for estimating evapotranspiration under conditions of minimal advection was formulated by Priestley and Taylor (1972). This model assumes the proportionality between the evaporation rate and available energy through a coefficient α, known as the Priestley-Taylor factor (Priestley and Taylor, 1972). This factor mitigates the uncertainties associated with the canopy resistance (Xu et al., 2021) and omits the aerodynamic term featured in the Penman-Monteith method (Pereira, 2004). Empirical findings suggest that the Priestley-Taylor parameter (α) is intricately linked to various physical factors influencing evapotranspiration (Pereira, 2004). Nevertheless, in addition to the requisite for local parameterization of α (Priestley and Taylor, 1972; Pereira, 2004), the model’s efficacy in semiarid regions remains variable (Xiaoying and Erda, 2005; Shahidian et al., 2012; Kustas et al., 2022).

In 1985, Shuttleworth and Wallace developed a physical process-based model to address the evapotranspiration of sparse crops. This model presupposes that total evapotranspiration encompasses the evapotranspiration from both the soil surface and vegetation canopy, considering the coupled effects of vapor and energy exchanges between the soil surface and vegetation canopy (Shuttleworth and Wallace, 1985). Treating sparse crops using the same conceptual framework as the Penman-Monteith method — a two-component system governed by energy balance and aerodynamic principles (Stannard, 1993) — the model shares analogous limitations with the Penman-Monteith method, particularly significant uncertainties in the computation of canopy and soil surface resistances (Chen et al., 2022).

The development of remote sensing methodologies, facilitated by the aerial or satellite imaging and advancements in image processing, has facilitated the computation of evapotranspiration as a residual of the surface energy balance (McShane et al., 2017). These methodologies employ an analytical approach to estimate evapotranspiration based on physically derived models that combine ground-based and remote sensing data (Courault et al., 2005).

Surface energy balance (SEB) models can be categorized into two categories:1. Single-source energy balance models analyze vegetation and soil within a unified energy budget, exemplified by the Surface Energy Balance Algorithm for Land (SEBAL) (Bastiaanssen et al., 1998) or the Mapping Evapotranspiration at High Resolution with Internalized Calibration (METRIC) (Allen et al., 2007). Within this context, Mallick et al. (2014) proposed an alternative big-leaf method, that eliminates the necessity for the parametrization of surface and aerodynamic conductances, achieved by integrating the radiometric surface temperature into the Penman-Monteith equation. The Mallick et al. (2014) surface temperature-initiated closure (STIC) method, driven by surface temperature, air temperature, relative humidity, net radiation, and ground heat flux, facilitates the decomposition of evapotranspiration into its constituent components (Mallick et al., 2014).

2. Dual-source energy balance models analyze vegetation and soil energy budgets independently, encompassing models such as Two-Source Energy Balance (TSEB) model (Norman et al., 1995; Kustas and Norman, 1999), Atmosphere-Land Exchange Inverse (ALEXI) (Anderson et al., 1997), and clumped models accounting with the clumping of vegetation to estimate evaporation from soil and sparse crops (Brenner and Incoll, 1997).

Two-source energy balance models provide more accurate estimations of evaporation from crops with more or less extensive bare surfaces, e.g. orchards, whereas single-source energy balance models are most suited for estimating transpiration from vegetated surfaces (Tang et al., 2013). The use of the TSEB model under the advection of hot dry air masses from surrounding non-irrigated areas necessitates modifications to accurately estimate ET under such conditions, as well as to ascertain the partitioning between evaporation and transpiration (Kustas et al., 2022).

Although, the inclusion of resistance terms remains imperative in the Penman-Monteith equation to elucidate the conjoined impacts of water stress and stomatal resistance on the transfer of heat and water flux (Alves and Pereira, 2000; Schymanski and Or, 2017; Zhao et al., 2020). This necessity is observed not only in other micrometeorological methodologies such as those articulated by Shuttleworth and Wallace (1985) but also in residual methods embedded within the energy-balance equation, exemplified by models like TSEB (two-source energy balance model) introduced by Norman et al. (1995).

Noteworthily, to devise accurate irrigation strategies, the use of simple and non-destructive plant-based methods is essential. In this context, vineyard evapotranspiration is chiefly governed by factors such as stomatal conductance to water vapor (g_sw_), net solar radiation (R_n_), air vapor pressure deficit (VPD), air temperature (T_air_) and wind speed (U) (Jarvis and McNaughton, 1986). Grapevine water status is significantly affected by soil moisture content, which governs plant water supply, and by leaf transpiration, which controls plant water loss (Dry and Loveys, 1999; Buckley, 2017; Levin et al., 2019). Both these factors influence the regulation of g_sw_ (Chaves et al., 2010; Costa et al., 2012; Buckley and Mott, 2013; Buckley, 2017; Levin and Nackley, 2021). Several studies aiming to identify water stress thresholds in grapevines have focused on using g_sw_ as a key indicator, especially for deficit irrigation strategies (Jones, 2004). However, factors such as genotype, leaf distribution within the canopy, exposure to sunlight, and high spatio-temporal variability within the vineyard have constrained the direct use of g_sw_ (Fernández and Cuevas, 2010; Bota et al., 2016).

Canopy conductance to water vapor (g_c_) plays a pivotal role in regulating water and heat exchange within the soil-plant-atmosphere continuum. It represents one of the most responsive variables to water stress in grapevines. The value of g_c_ lies in its ability to elucidate the division of R_n_ between latent heat and sensible heat fluxes (Monteith, 1995), thereby influencing plant transpiration. As the canopy acts to stabilize the considerable variability in g_sw_ across individual leaves, g_c_ offers a suitable spatio-temporal measure of stomatal response to soil water and atmospheric conditions within the boundary layer (Avissar, 1993; Damour et al., 2010; Fuentes et al., 2012; Wu et al., 2022). Consequently, it allows the identification of temporal and spatial patterns of water stress in the vineyard (Zhai et al., 2020).

While g_c_ can be estimated by inverting the Penman-Monteith equation (Monteith and Unsworth, 2013), the complexity and feasibility constraints of its parameterization have led to the proposal of alternative methods for estimating g_c_ in grapevines. These models are categorized based on how they aggregate canopy properties and account for atmospheric properties that influence the canopy. The simplest, big-leaf (BL) models, scale the leaf g_sw_ to the entire canopy, treating it as a single leaf (Baldocchi et al., 1967; Todorovic, 1999; Zhang et al., 2008). On the other hand, the two leaf models consider the contributions of the sunlit and shaded leaf fractions, scaling them using the associated g_sw_ weighted by their respective fractions of the leaf area index (LAI) (Leuning et al., 1995; Wang and Leuning, 1998; Ding et al., 2014). Lastly, the multilayer models, assume spatial independence of leaf gas exchange properties and consider atmospheric properties’ variation across canopy layers (Baldocchi and Meyers, 1998; Chen et al., 1999).

This article evaluates the accuracy of a biophysical model based on a BL approach that utilizes g_sw_ from leaves with contrasting sun exposure, scaled to the canopy size by the LAI, along with variables such as R_n_ and air VPD, to estimate g_c_. The model’s validity is established through comparison with g_c_ measurements derived from the gold-standard inverse Penman-Monteith method (Monteith and Unsworth, 2013). The outcomes reveal that the proposed model, grounded in both plant and atmospheric variables, proficiently predicts g_c_, thus positioning it as a promising indicator for assessing grapevine stress.

## 2 Material and methods

### 2.1 Location and experimental layout

Field experimentation was carried out in an experimental vineyard known as Herdade do Esporão, located in Reguengos de Monsaraz, within the Alentejo wine growing region in southern Portugal (latitude 38^◦^ 23′ 55.00′′ N; longitude 7^◦^ 32′ 46.00′′ W). For this study, the red *Vitis vinifera* cv. Tempranillo variety, grafted onto 1103 Paulsen rootstock, was selected. The vineyard encompasses a density of 2220 vines per hectare, with a spacing of 1.5 meters within rows and three meters between rows, all north-south oriented. The vines are trained on a vertical shoot positioning system and are uniformly pruned, maintaining 15 to 16 buds per vine in a bilateral Royat cordon system. The soil, identified as an Eutric Cambisol (CM), has a depth of approximately one meter and features a silty-clay-loam texture. Standard cultural practices prevalent in the region were employed. Irrigation followed the vineyard owners’ practices, with an average application of 140 mm of water from the berry set to harvest (approx. 30% crop evapotranspiration, ET_c_).

Data collection was carried out on the 20^th^ of August 2019, the 29^th^ of July 2020, the 8^th^ and 15^th^ of July 2021, and the 12^th^ of August 2021, from 9:00 am to 7:00 pm, as detailed in section 2.3. For this purpose, 20 vines evenly distributed were selected along two adjacent rows, with 10 vines in each row. This arrangement allowed for a thorough examination of the impact of solar radiation, as it enabled the study of both sides of the canopy (east and west) under varying incident radiation conditions.

To ensure the measurements’ accuracy, and to mitigate any potential influence from soil water evaporation, measurements were carried out at least five days after the most recent irrigation, thus ensuring that fluctuations in the soil surface’s water content did not confound the readings. This precaution was crucial to guarantee that, under these controlled conditions, no soil evaporation was observed. To confirm the negligible impact of soil evaporation, the soil water content was continuously monitored beneath the grapevine canopy in the top 10 cm of the irrigated soil portion. To this effect, the readings of two ML3 Thetaprobe sensors (Delta-T devices, Cambridge, UK) were collected every 15 minutes and registered into a datalogger (CR1000, Campbell Scientific, Inc., Logan, UT, USA) for convenient analysis. In addition to soil measurements, the vines water condition was evaluated by measuring the pre-dawn leaf water potential (Ψ_PD_), by using a Scholander type pressure chamber on each measurement day. This measure provides valuable insights into the water status of the grapevines.

### 2.2 Biophysical model proposal to predict canopy conductance to water vapor. Theoretical development

The central objective of this study is to develop an effective grapevine canopy conductance model capable of accurately representing the grapevine’s response to water scarcity, increased solar irradiance, and high atmospheric water demand. The modelling approach encompasses the considerations and assumptions described below.

In the context of the Mediterranean climate, the summer season is characterized by reduced precipitation levels, abundant solar radiation, high air temperatures, and elevated atmospheric evaporative demand. Consequently, grapevines experience pronounced water and heat stress during this period (Chaves et al., 2016). To optimize the utilization of available solar radiation and facilitate vineyard management, grapevines are typically arranged in rows. This configuration results in a low canopy profile, which significantly increases the portion of soil directly exposed to evaporation (Costa et al., 2019; Costa et al., 2023). As the grapevines reach their maximum vegetative growth during the veraison stage, soil water availability rapidly diminishes, thus requiring irrigation intervention. Drip irrigation systems are typically positioned beneath the grapevine canopy to meet the plants’ water requirements. Moreover, irrigation often falls short of meeting the actual crop needs, particularly with deficit irrigation strategies, which limit soil water evaporation. Accurately partitioning evapotranspiration into its constituent elements, plant transpiration and soil water evaporation, becomes especially relevant in arid environments, making it a crucial source of information for water management decisions (Kool et al., 2014).

The primary regulation of vine transpiration is through stomata, which responds to changes in atmospheric demand and soil water availability, as explained by Chaves et al. (2010); Chaves et al. (2016). Consequently, a simplification of the Penman-Monteith approach has been employed, assuming a similarity between canopy resistance and the integration of leaf stomatal resistances under dry conditions (Shuttleworth and Wallace, 1985). Analogous to Ohm’s law, this model considers the proportional relationship between canopy conductance and the available energy for evaporation (R_n_), as well as the inverse relationship with stomatal resistance (Lhomme, 1991; Alfieri et al., 2008). Additionally, the model accounts for changes in canopy conductance induced by air vapor pressure deficit (VPD) (Wang and Leuning, 1998; Lu et al., 2003; Chaves and Oliveira, 2004; Buckley, 2005; Rogiers et al., 2012; Buckley and Mott, 2013; Klein, 2014; Sperry et al., 2017; Luo et al., 2018; Zhu et al., 2022; Zhong et al., 2023). VPD is closely linked to climatic conditions, and variations in stomatal conductance (g_sw_) responses to VPD may be associated with varying levels of atmospheric dryness (Rogiers et al., 2012; Grossiord et al., 2020; Liu et al., 2020; Zhong et al., 2023). Furthermore, VPD accounts for the difference between the actual moisture content in the air and the moisture it could hold at saturation (Allen et al., 1998), thereby affecting soil and canopy evaporation. As a result, a comprehensive model that considers all parameters governing energy exchange and the corresponding latent heat flux has been developed. This model estimates canopy conductance, g_c est_, based on factors such as energy inputs, R_n_, atmospheric demand, VPD, leaf stomatal conductance, g_sw_, and leaf area index, LAI. This approach accounts for the spatial independence of leaf gas exchange characteristics within sunlit and shaded leaves, while also considers the distinct atmospheric conditions present in these various canopy layers.

Thus, the proposed model to estimate canopy conductance, g_c est_, is calculated as follows:


(1) $$g_{c\;est}=f\;\left(LAI,\;g_{sw},R_{n},VPD\right)=LAI.{\left(g_{sw}.\frac{R_{n}}{VPD}\right)}^{0.5}\left(m.s^{-1}\right)$$

where LAI is the dimensionless leaf area index, g_sw_ is the leaf stomatal conductance measured in *m.s^−1^* for both sunlit and shaded leaves, R_n_ stands for the net solar radiation at the canopy level, measured in *MJ.m^−2^.h^−1^*, and VPD represents the air vapor deficit, measured in *Pa*. Air VPD is calculated from relative humidity, RH, and air temperature, T_air_, by applying the following Equations (2-5) defined by Allen et al. (1998):


(2) $$VPD=\;e_{s}-e_{a}\left(Pa\right)$$

where


(3) $$e_{a}=\;e_{0}\left(T_{air}\right).\frac{RH}{100},$$

(4) e0(Tair)= 0.6108×e[(17.27.Tair)(Tair+237.3)]

and


(5) $$e_{s}=\;\frac{\left[e_{0}\left(T_{min}\right)+e_{0}\left(T_{max}\right)\right]}{2}$$

being T_min_ and T_max_ the minimum and maximum air temperature (*°C*) registered for a given period, respectively.

The proposed model is designed to maintain the dimensions of the dependent variable g_c est_ [*L.T^−1^*], where *L* represents length, and *T* represents time. This modelling process accounts for the influence of environmental factors and the regulation of stomata on grapevine leaves, thus contributing to the overall behavior of the canopy surface conductance.

### 2.3 The reference method, the inverted Penman-Monteith equation

The Penman-Monteith method (Monteith and Unsworth, 2013) is a widely recognized tool to estimate canopy conductance (g_c_), so it is used as gold-standard reference to assess goodness of the biophysical model proposed in this paper.

Motivated by the absence of significant soil evaporation, as explained in section 2.1, the bulk surface conductance (g_s_) was assumed to be solely determined by the canopy conductance (g_c_) (Allen et al., 1998). As a result, the observed canopy conductance (g_c obs_), used to validate the proposed model, was calculated from the inverted Penman -Monteith equation, according to the formulation proposed by Lhomme et al. (2012), as follows:


(6) $$g_{c\;obs}=\frac{\gamma .\lambda .E_{c}.g_{a}}{\Delta .R_{n}+k_{t}.\rho .C_{p}.VPD.g_{a}-\lambda .(\Delta +\gamma ).E_{c}}\left(m.s^{-1}\right)$$

where:

- *R_n_* is net radiation (*MJ.m^−2^.h^−1^*).
- *k_t_* is a time unit conversion (3600 *s.h^−1^*).
- *λ* is the latent heat of water vaporization (2.45 *MJ.kg^−1^*).
- *C_p_* is the dry air specific heat at constant pressure (0.001013 *MJ.kg^−1^.°C^−1^*).
- *VPD* is air vapor pressure deficit as defined in Equation 2.
- *E_c_* is canopy transpiration in *mm.h^−1^* units.
- *γ*  is the psycrometric constant, calculated according to Allen et al. (1998) by the Equation 7:

(7) γ=Cp.Pε.λ(kPa.oC−1)

where *ε* is the ratio of the molecular weight of water vapor/dry air (dimensionless and equal to 0.622), *P* is the atmospheric pressure, depending only on the local elevation above sea level (220 m in this study), and equal to 98.726 *kPa*, and *λ* and *C_p_* are the previously mentioned latent heat of vaporization and dry air specific heat at constant pressure, respectively.
•*g_a_* is the aerodynamic conductance, as defined by Equation 8 (Granier et al., 2000):

(8) $$g_{a}=\frac{k^{2}\cdot U_{z}}{ln\left[\left(z_{m}-d\right)/z_{om}\right].ln\left[\left(z_{m}-d\right)/z_{ov}\right]}\left(m.s^{-1}\right)$$

where *k* is the von Karman’s constant (0.41), *Uz* is wind speed at height *z_m_* (*m.s^−1^*) (Monteith and Unsworth, 2013), *z_m_* is the height of wind speed and humidity measurements (3 m in this study), *d* is the zero-plane displacement height (*m*), and *z_om_* and *z_ov_* are the roughness lengths governing transfer of momentum and water vapor (*m*). The quantities *d*, *z_om_* and *z_ov_* were estimated using *d* = 2*h*/3, *z_om_* = 0.123∙*h* and *z_ov_* = 0.1*∙z_om_*, where *h* is canopy height (averaged as 1.7 m in this study) (Allen et al., 1998).
•*Δ* is the vapor pressure curve’s slope, as defined by Equation 9 Allen et al. (1998):

(9) Δ =4098×e0(Tair)(Tair+237.3)2

where $e_{0}\left(T_{air}\right)$ is the saturation vapor pressure at air temperature T_air_, computed in *kPa* according to the Equation 4.
•*ρ* is air density, calculated according to Equation 10:

(10) ρ= P[1.01×(Tair+273.3)×R]

where *P and T_air_* are the previously mentioned atmospheric pressure and air temperature, and *R* is the specific gas constant (287 *J.kg^−1^.K^−1^*).

### 2.4 Field and reference measurements

#### 2.4.1 Climate and grapevine water status

The climate is of Mediterranean type, with hot and dry summers and mild rainy winters. Rainfall accumulated from winter to flowering ranged from 338 to 535 mm between 2019 and 2021. The maximum precipitation recorded in the three years from berry set to full ripening (June to August) was 13.4 mm in 2021 (**Table 1**). There were no recorded instances of rain events during the analyzed period, and none of the measurements were taken under cloudy conditions.

**Table 1:** Rainfall figures within the experimentation window: rainfall registered during the grapevine dormancy period, which spans from October (Oct) to December (Dec) of the previous vintage year [Year (i-1)]; rainfall registered during the period from dormancy to flowering (January (Jan) to May); rainfall registered during the period from berry development to grape ripening [June (Jun) to August (Aug)] of the current vintage year [Year (i)]; and accumulated rainfall from October of the vintage previous year to August (Aug).

	Rainfall (mm)			
	Year (i−1)	Year (i)		Accum
***Year (i)***	*Oct-Dec*	*Jan-May*	*Jun-Aug*	*Oct-Aug*
***2019***	204.4	133.6	3.4	341.4
***2020***	226.0	239.0	1.2	466.2
***2021***	249.8	285.4	13.4	548.6

Overall, the predawn leaf water potential (Ψ_PD_) measurements consistently indicated moderate to severe water stress, which is considered adequate for producing high-quality vintages (Deloire et al., 2005; van Leeuwen et al., 2009). Moreover, the readings showed a contained maximum variation of 0.1*Mpa* between the minimum and maximum observed values (**Table 2**).

**Table 2:** Predawn leaf water potential (Ψ_PD_) figures measured at the predawn on the 20^th^ of august 2019, the 29^th^ of July 2020, the 8^th^ and 15^th^ of July 2021, and the 12^th^ of August 2021.

DAY	Ψ_PD_ (*MPa*)	
	Mean	SE
20-08-2019	−0.60	0.012
29-07-2020	−0.55	0.017
8-7-2021	−0.50	0.010
15-7-2021	−0.50	0.010
12-8-2021	−0.60	0.015

Data are presented by the means (Mean) and standard error of the mean (SE) of 5 repetitions by day.

#### 2.4.2 Field measurement of key variables

This section describes the in-the-field measurement of key variables involved in the calculation of the proposed biophysical model (section 2.2, Equation 1), and of the reference inverted Penman-Monteith equation (section 2.3, Equation 6).

Under the experimental conditions described in section 2.1, where soil evaporation was found to be negligible, actual crop evapotranspiration (ET_c act_) served as a suitable proxy for vine transpiration (E_c_). The measurements of E_c_ were conducted concurrently with those of wind speed, *U*, at a high temporal resolution (every 1/10 *s*), this to capture fine-scale variations in transpiration dynamics. The equipment employed to this end, was composed of an eddy covariance system comprising a fast-response, 0.1 Hz, open-path CO2/H2O analyzer (LI-6500 DS, LI-COR Inc., Lincoln, NE, USA), along with a 3D sonic anemometer (Gill Windmaster Pro, Gill Instruments Limited, Hampshire, UK). For experimental requirements, the eddy covariance system was installed over the top of the canopy, concretely at a height of 3.0 m above the ground surface. The acquired data underwent processing using EddyPro software v7.0.6 (LI-COR Inc., Lincoln, NE, USA) for the purpose of quality testing and analysis. The data collection setup was designed to cover a fetch distance of at least 300 m, aligned with the prevailing northerly winds, thereby ensuring comprehensive measurement of fluxes within the designated area of interest. The utilization of EddyPro software played a crucial role in quality assessment and analysis, revealing that over 90% of the daily data flux within the analyzed dataset exhibited an energy balance closure greater than 0.95.

Air temperature (T_air_) and relative humidity (RH) were recorded using a thermohygrometer (CS215-PWS, Campbell Scientific, Inc., Logan, UT, USA), which was positioned at a height of 2.0 m above the ground, and thus over the canopy. Additionally, net radiation (R_n_) over the canopy was quantified using a net radiometer (NR2, Delta-T Devices, Cambridge, UK), also positioned above the canopy. All these soil and meteorological sensors were integrated into a datalogger (CR1000, Campbell Scientific, Inc., Logan, UT, USA), which addressed data saving at every minute.

The Leaf area Index (LAI) of grapevines was determined using a non-destructive allometric method applied to the 20 grapevines selected for the research. The method by Lopes and Pinto (2005) was utilized to compute the entire area of grapevine leaves. Thus, LAI was estimated from the overall leaf area divided by the area occupied by each individual vine, being measured on four occasions: the 21^st^ of August 2019, the 24^th^ of July 2020, the 14^th^ of July 2021, and the 16^th^ of August 2021. Water vapor gas exchange was assessed by hourly measurements of stomatal conductance (g_sw_) on mature leaves from both sides of the canopy, using a steady-state porometer (LI-1600, LI-COR, Lincoln, NE, USA).

**Table 3** summarizes key information related to the described variables measured in the field.

**Table 3:** Summary of field measurements.

Variable measurements		
Canopy transpiration (*E_c_*)		
***Source***		LICOR Model LI-6500 DS, (LI-COR Inc., Nebraska USA) eddy covariance system. Measured when soil evaporation was found to be negligible.
***Periodicity***		*Every 1/10 seconds*
***Units***		*mm.h^−1^*
Net radiation (*R_n_*)		
***Source***		Net radiometer, model NR2, (Delta-T Devices, Cambridge, UK) connected to a datalogger (CR1000, Campbell Scientific, Inc., Logan, UT, USA)
***Periodicity***		*Every minute*
***Units***		*MJ.m^−2^.h^−1^*
Air Temperature (*T_air_*)		
***Source***		Thermohygrometer Model CS215-PWS (Campbell Scientific, Inc., Logan, UT, USA), connected to a datalogger (CR1000, Campbell Scientific, Inc., Logan, UT, USA)
***Periodicity***		*Every minute*
***Units***		*°C*
Relative Humidity (*RH*)		
***Source***		Thermohygrometer Model CS215-PWS (Campbell Scientific, Inc., Logan, UT, USA), connected to a datalogger (CR1000, Campbell Scientific, Inc., Logan, UT, USA)
***Periodicity***		*Every minute*
***Units***		*%*
Wind speed (*U*)		
***Source***		3D sonic anemometer (model GillWindmaster Pro, Gill Instruments Limited, Hampshire, UK)
***Periodicity***		*Every 1/10 seconds*
***Units***		*m.s^−1^*
Stomatal conductance to water vapor (g_sw_)		
***Source***		Steady state porometer (Model LI-1600, LI-COR Lincoln, NE, USA)
***Periodicity***		*Hourly from 9 am to 7 pm*
***Units***		*m.s^−1^*
Leaf area index (*LAI*)		
***Source***		Non-destructive allometric method applied to compute the entire area of grapevine leaves (Lopes and Pinto, 2005). The LAI was estimated from the overall leaf area divided by the area occupied by each individual vine.
***Periodicity***		On the 21^st^ of August 2019, the 24^th^ of July 2020, the 14^th^ of July 2021, and the 16^th^ of August 2021
***Units***		*m^2^∙m^−2^*

This table provides a comprehensive summary of the field measurements used in the study, including the measurement name, data collection periodicity, units of measurement, and the source equipment for each measurement.

### 2.5 Implementation and evaluation

#### 2.5.1 Implementation of the proposed biophysical model. Data processing and analysis

In order to assess the accuracy of the proposed biophysical model (Equation 1), a dataset was built including the implied variables measured in the field and included in **Table 3**. Namely:

- Leaf area index (LAI, *m^2^.m−^2^*).
- Net solar radiation (R_n_, *MJ.m⁻^2^.h−^1^*).
- Air vapor pressure deficit (VPD, *Pa*), calculated according to Equation 2 from the measured variables RH and T_air_ (*°C*).
- Stomatal conductance to water vapor (g_sw_, *mol.m⁻^2^.s⁻^1^*).

To make the proposed method suitable for scaling to canopy dimensions, note that LAI was considered as a factor in Equation 1. Notwithstanding, LAI measured values of the selected vines throughout the three seasons covered during the experimentation, was always in the range 1.91 to 2.0 *m^2^.m^−2^*, so a mean value of 1.96 *m^2^.m^−2^* was used in practice.

To ensure consistency in modelling and facilitate unit compatibility, the units of g_sw_ were converted to *m.s⁻^1^* by using the molar density of air (*mol.m⁻^3^*). On the other hand, to mitigate the influence of very low net solar radiation (R_n_) on stomatal opening, data collected before 10 am and after 6 pm, when R_n_ was below 0.60 *MJ.m⁻².h⁻¹*, were excluded. This exclusion respects the principles established by Granier et al. (2000). No outliers were identified using the interquartile range (IQR) method with a cut-off of 1.5 times the IQR. Consequently, the final dataset included 550 measurements of g_sw_, R_n_, and air VPD, along with the mean value of LAI.

#### 2.5.2 Methodology for model evaluation

The evaluation of the model involved fitting the estimated canopy conductance values, g_c est_, given by the model proposed in this study (Equation 1), to the observed canopy conductance values, g_c obs_, obtained by using the inverse Penman-Monteith equation (Equation 6). Thus, the predictive potential of the model is firstly quantified by using the Pearson’s coefficient of correlation (R). Additionally, the following accuracy measures were used:

1. The Root-Mean-Square Error (RMSE), measuring the overall deviation between predicted and observed canopy conductance values, defined by Equation 11 as follows:

(11) $$RMSE=\;\sqrt{\frac{\sum_{i=1}^{n}{\left(g_{c\;est}^{i}-\;g_{c\;obs}^{i}\right)}^{2}}{n}}$$

2. The Mean Absolute Error (MAE), measuring the absolute error between estimated and observed g_c_, was determined using Equation 12:

(12) $$MAE=\frac{\sum_{i=1}^{n}\left|g_{c\;est}^{i}-g_{c\;obs}^{i}\right|}{n}$$

3. The Relative Error (|E|), quantifying the estimation error as a percentage of the observed value, was determined by Equation 13:

(13) $$\left|E\right|=\frac{\left|\sum_{i=1}^{n}\left(g_{c\;est}^{i}-g_{c\;obs}^{i}\right)\right|}{\sum_{i=1}^{n}g_{c\;obs}^{i}}x100$$

In the presented formulas, $g_{c\;est}^{i}$ is the *i*-th g_c_ value estimated by the proposed model formulated in Equation 1, from the dataset of *n=550* elements, and $g_{c\;obs}^{i}$ is the *i*-th observed g_c_ value given by the Penman-Monteith equation, calculated on the same dataset.

Model Evaluation processes were implemented using Matlab R2021b (The Mathworks Inc.).

## 3 Results and discussion

### 3.1 Characterization and variance of canopy conductance predictors

**Figure 1:**
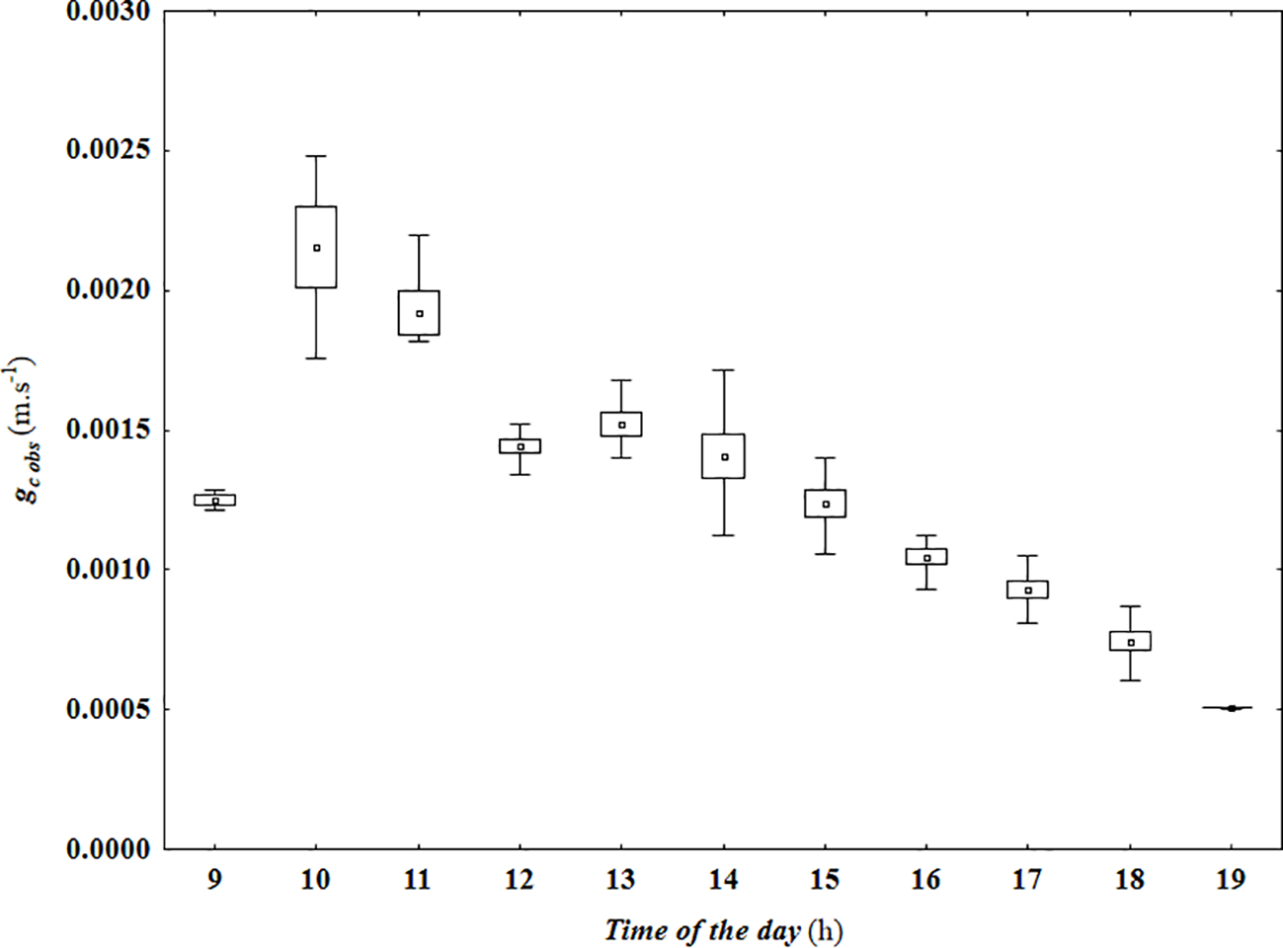
Hourly variation of canopy conductance to water vapor (g_c obs_, m.s^−1^), calculated using the inverted Penman-Monteith equation (Equation 6), using the variables measured at the experimental plot. Data are represented by their mean (▫), 95% confidence interval (□), and respective non-outlier range (┬).

**Figure 2:**
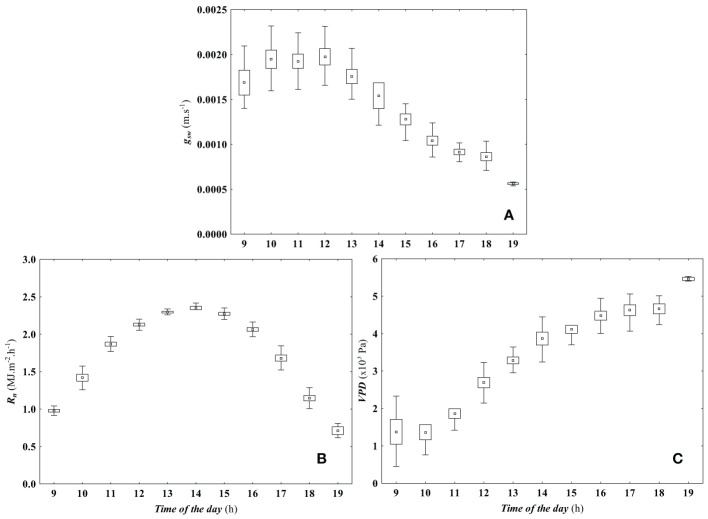
Hourly variation of canopy conductance predictors. The figure shows the hourly variation of key canopy conductance predictors, including **(A)** stomatal conductance to water vapor (g_sw_, m.s^−1^), **(B)** net solar radiation above the canopy (R_n_, MJ.m^−2^.h^−1^), and **(C)** air vapor pressure deficit (VPD, Pa). The figure illustrates the diurnal patterns of these predictors during the experimental period. Data are represented by their mean (▫), 95% confidence interval (□), and respective non-outlier range (┬).

**Figure 1** represents the hourly variation of canopy conductance to water vapor, g_c obs_, calculated with the Penman Monteith’s method (Equation 6). As it can be observed, it exponentially increased from sunrise to a peak of approximately 0.0028 m*.s^−1^* at 10 am, and then gradually decreased until the end of the day to a minimum of 0.0005 m*.s^−1^* at 7 pm. Hourly variation of the biophysical variables used in the proposed model formulated in Equation 1, measured at the experimental plot during defined period, are presented in **Figure 2**; namely: stomatal conductance to water vapor (g_sw_, *m.s^−1^*), net solar radiation above the canopy (R_n_, *MJ.m^−2^.h^−1^*), and air vapor pressure deficit (VPD, *Pa*). The plot shows an inverted trend of g_sw_ to the daily variation of air VPD. Since no highly severe water stress was imposed to the vines, the maximum g_sw_ value was attained between 10:00 and 12:00 hours, when stomata were not limited either by the available R_n_ (Jones et al., 2002) neither air VPD (Rogiers et al., 2012; Zhong et al., 2023).

**Figure 3:**
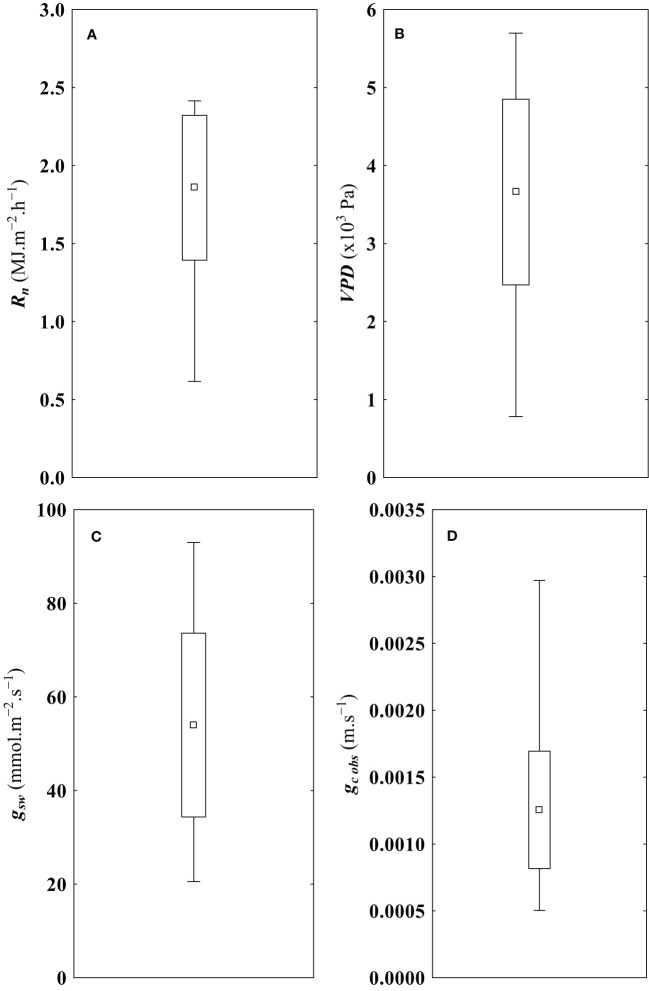
Characterization of model predictors and response variables. **(A)** Net solar radiation above the canopy (R_n_), **(B)** air vapor pressure deficit above the canopy (VPD), **(C)** leaf stomatal conductance to water vapor (g_sw_), and **(D)** canopy conductance calculated by Penman Monteith method using canopy transpiration (E_c_) registered on an eddy covariance flux tower as input (g_c obs_). The data are characterized by the mean (▫), the standard deviation range ((□)) and the respective minimum and maximum value (┬) of each variable. (N = 550).

**Figure 3** provides box and whisker plots of the biophysical variables used in the proposed model. The statistical characterization of g_c obs_, is also shown, which revealed a tight clustering of the data around the mean. A subsequent analysis of g_c obs_, also showed a strong and significant correlation with the biophysical variables used in the proposed model (see **Table 4**). Similar results were referred by Monteith (1995); Wang and Leuning (1998); Irmak et al. (2008); Lhomme et al. (2012); Ding et al. (2014) or Wu et al. (2022). As indicated in **Table 4**, when considering all the variables used to calculate g_c obs_, it is worth noting that only measured wind speed, *U*, exhibited a weak correlation with the observed changes in canopy conductance (g_c obs_). This observation is consistent with findings previously reported by Lhomme (1991). Consequently, wind speed was not included as a predictor in the proposed model.

**Table 4:** Correlation matrice between the reference canopy conductance (g_c obs_) calculated with the inverted Penman-Monteith equation, and field data measured at the experimental plot: net solar radiation above the canopy (R_n_), air vapor pressure deficit (VPD), stomatal conductance to water vapor (g_sw_), and wind speed (U).

	g_c obs_ (m.s^−1^)			
	Rn (*MJ.m^−2^.h^−^* ^1^)	VPD (Pa)	g_sw_ (m.s^−1^)	U (m.s^−1^)
20 Aug 2019 *sig.*	0.833 ***	−0.962 ***	0.773 ***	−0.282 *n.s.*
29 Jul 2020 *sig.*	0.858 ***	−0.832 ***	0.994 ***	−0.221 *n.s.*
8 Jul 202 *sig.*	0.866 ***	−0.811 ***	0.849 ***	−0.298 *n.s.*
15 Jul 2021 *sig.*	0.831 ***	−0.871 ***	0.963 ***	−0.354 *n.s.*
12 Aug 2021 *sig.*	0.893 ***	−0.866 ***	0.969 ***	−0.265 *n.s.*

Significance levels: n.s. (not significant); * (significant differences at a 90% confidence level); **(significant differences at a 95% confidence level); *** (significant differences at a 99% confidence level).

Since no precipitation fell during the measurement periods, no evaporation was registered from the soil between the vine rows. Furthermore, no fluctuations were found in terms of water content in the soil surface under the vines either, thus corroborating the absence of evaporation on the measurement days. Under these conditions, actual crop evapotranspiration, ET_c act_, was strongly related to vine transpiration, E_c_, and strongly controlled by canopy conductance, g_c_ (**Table 5**). Similar results were presented by Lu et al. (2003).

**Table 5:** Correlation matrice between the reference canopy conductance (g_c obs_) calculated with the inverted Penman-Monteith equation, and the field data vine transpiration (E_c_) measured at the experimental plot.

	*VPD ≤ 3 kPa*	*VPD > 3 kPa*
20 Aug 2019 *sig.*	−0.962 ***	0.895 ***
29 Jul 2020 *sig.*	−0.832 ***	0.985 ***
8 Jul 2021 *sig.*	−0.811 ***	0.972 ***
15 Jul 2021 *sig.*	−0.871 ***	0.930 ***
12 Aug 2021 *sig.*	−0.866 ***	0.988 ***

Significance levels: n.s. (not significant); * (significant differences at a 90% confidence level); **(significant differences at a 95% confidence level); *** (significant differences at a 99% confidence level).

The data is categorized into two groups based on the vapor pressure deficit (VPD) conditions: VPD ≤ 3 kPa (morning) and VPD > 3 kPa (afternoon).

**Figure 4:**
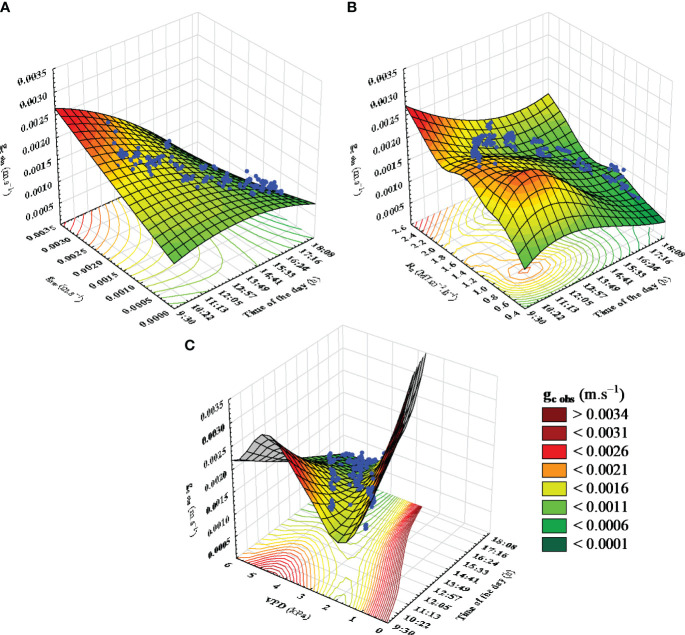
Hourly response of reference canopy conductance (g_c obs_) to model predictors (R_n_, VPD, g_sw_). The figure illustrates the variation in reference canopy conductance (g_c obs_) throughout a typical day in response to **(A)** leaf stomatal conductance (g_sw_), **(B)** net solar radiation above the canopy (Rn), and **(C)** air vapor deficit (VPD). Response surfaces were fitted by Distance-Weighted Least Squares method.

On a Typical Mediterranean summer day, there is an abundance of solar radiation and high temperatures, along with low relative humidity, resulting in elevated air VPD. Under these circumstances, grapevines undergo significant water and heat stress from midday until the day’s end (Chaves et al., 2016). **Figure 4** depicts how g_c obs_ fluctuates during a typical Mediterranean day in response to g_sw_, R_n_, and air VPD. Following the available solar radiation, g_c obs_ increased rapidly after sunrise, reaching a maximum of approximately 0.003 m*.s* ^−1^ at 10 a.m (**Figure 4B**). As also mentioned by Rogiers et al. (2012), it is noteworthy that air VPD did not appear to limit g_sw_ during this period (**Figures 2A, C** and **4A**). Subsequently, g_sw_ began to decrease as air VPD increased (**Figures 2A, C**, **4A**). In addition, g_c obs_ started a progressive decrease until reaching 0.0005 m*.s* ^−1^ at 6 pm (**Figures 1**, **4A–C**).

### 3.2 Model performance evaluation results

**Table 6** presents the accuracy measures of the proposed biophysical model (Equation 1) used to estimate canopy conductance (g_c est_). These quantitative accuracy metrics allows to assess the extent to which the model’s predictions align with those given by the reference method, i.e., the observed canopy conductance (*g_c obs_*) given by the inverted Penman-Monteith equation (Equation 6).

**Table 6:** Goodness-of-fit measures between the reference canopy conductance (g_c obs_), calculated with the inverted Penman-Monteith equation (Equation 6), and the estimated canopy conductance (g_c est_) given by the proposed biophysical model (Equation 1).

	RMSE	MAE	|E|
g_c_	1.45x10^−4^	9.45x10^−1^	7.5

These metrics encompass: root mean square error (RMSE), mean absolute error (MAE), and relative error (|E|). The metrics were calculated on the dataset of 550 samples (n = 550).

The model’s estimations demonstrate a high degree of agreement with the reference dataset obtained by calculating the inverted Penman-Monteith equation. Furthermore, the model’s metrics for residuals deviation, which evidence the discrepancies between the observed and estimated values of g_c est_, are notably lower than the minimum g_c obs_, in the context of the statistical profile represented in **Figure 3D**. It is important to emphasize that all goodness-of-fit metrics underscore the model’s robustness within the specific conditions of the study.

**Figure 5:**
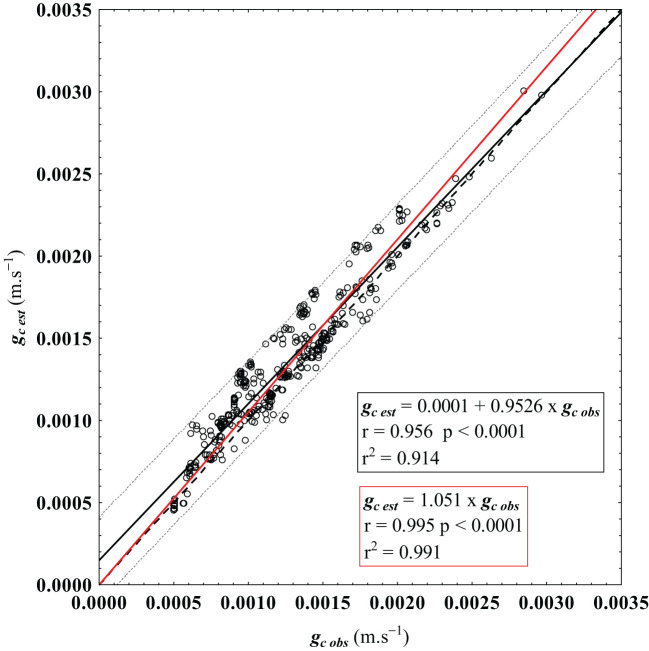
Least square regression (solid black line) between the reference g_c obs_ (observed), calculated from the inverted Penman-Monteith equation (Equation 6), and the estimated canopy conductance g_c est_, given by the proposed model (Equation 1). Confidence prediction interval at 95% is represented by the dotted lines. Additionally, a regression through the origin is shown in a solid red line, and the dashed line represents the 1:1 relationship, which indicates a perfect matching between the observed and estimated values.


**Figure 5** shows the scatterplot depicting the linear relationship between *g_c obs_* values and *g_c est_* values. As it can be checked, the obtained linear regression closely aligns with the 1:1 line, which indicates proper model fitting. Indeed, the estimated data exhibits a very high and statistically significant coefficient of correlation (r = 0.956, p<0.0001) with the observed values. Notably, at a 95% confidence level, most of the plotted data falls within the prediction interval. However, a tendency to overestimate the reference canopy conductance, particularly for smaller g_c obs_ values near the intercept point, can be noticed. To access the significance of the model’s overestimation near the intercept point (β_0_), a hypothesis test was conducted to determine whether the intercept of the least-squares regression between *g_c obs_* and *g_c est_*, could be considered null (β_0_ = 0). This null hypothesis (H_0_: β_0_ = 0) was tested using a *t*-Student’s statistic (Eisenhauer, 2003). The simple linear regression between the observed and estimated canopy conductance was defined by Equation 14 as:

(14) $$g_{c\;est}=\beta_{0}+\beta_{1}\cdot g_{c\;obs}+e_{i}$$

where β_0_ is the intercept, β_1_ is the slope, and e_i_ denotes the *i*-th residual.

The results showed that the null hypothesis (H_0_) cannot be rejected (p = 0.124), leading to the use of a regression through the origin, considering β_0_ = 0, to compare the model estimates with the reference values. This least square regression between g_c obs_ and g_c est_ (g_c est_=1.051∙g_c obs_) also exhibited a very high and statistically significant coefficient of correlation (r = 0.995, p<0.0001) between the observed and estimated g_c_ values (**Figure 5**).

**Figure 6:**
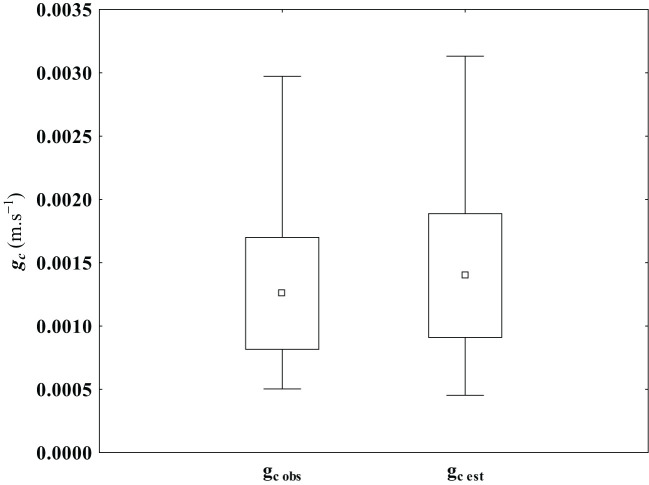
Comparison of observed (g_c obs_) and estimated (g_c est_) canopy conductance datasets. The datasets are characterized by their respective mean (▫), the standard deviation range (□) and the minimum and maximum value (┬). (N = 550).


The model’s ability to make accurate predictions is highlighted when we compare the estimated and observed canopy conductance. The biophysical model’s estimation of g_c est_ was, at most, approximately 10% lower than the minimum observed value (minimum g_c obs_ = 0.0005 m*.s^−1^*) (see **Figure 6**). Conversely, the model slightly overestimated the maximum observed canopy conductance by 4%. It’s worth noting that the overestimation for both the average and standard deviation remained within the 10% range, with observed values at 0.00126 and ±0.00044 *m.s^−1^*, respectively (**Figure 6**). The two datasets achieved comparable coefficients of variation, specifically 34.36% and 34.37% for the observed and estimated canopy conductance data, respectively. A Cohen’s measure for effects size (*d*) was applied to the mean differences between the observed and estimated canopy conductance. Cohen’s *d* measure was defined according to Cohen (1988), as:

(15) $$d=\frac{\bar{g_{c\;obs}}-\bar{g_{c\;est}}}{s_{p}}$$

where $\bar{g_{c\;obs}}$ is the mean value of the g_c obs_ measures, $\bar{g_{c\;est}}$ is the mean value of the g_c est_ measures, and *s_p_* is the pooled standard deviation, defined by:


(16) $$s_{p}=\sqrt{\frac{\left(N_{obs}-1\right).s_{obs}^{2}+\left(N_{est}-1\right).s_{est}^{2}}{N_{obs}+N_{est}-2}}$$

where N_obs_, N_est_, $s_{obs}^{2}\;$ and dd $s_{est}^{2}$ are in Equation 16 the number of samples and the variance of the g_c obs_ and g_c est_ datasets, respectively.

The calculated Cohen’s measure (Equation 15) was compared to Cohen’s standards for very small (*d*<0.2), small (0.2≤*d*<0.5), medium (0.5≤*d*<0.8), and large (*d*≥0.8) effect sizes (Cohen, 1988; Sawilowsky, 2009). Results revealed a small difference between the mean of g_c obs_ and g_c est_, equal to 21.7% of the standard deviation of the reference g_c obs_ (Lakens, 2013). Additionally, according to Cohen’s criteria, only 15.8% of the g_c est_ exhibited no overlap with the reference g_c obs_ data (Cohen, 1988).

These results emphasize the proposed biophysical model’s capability to estimate vine canopy conductance under the stressful conditions of this study. Indeed, they highlight the model’s effectiveness in capturing the impact of climatic factors such as net solar radiation, vapor pressure deficit, and leaf stomatal conductance on canopy conductance. This finding aligns with the conclusions of other researchers, who have emphasized the significant impact of stomata on gas exchange, affecting both leaf-level transpiration (Chaves et al., 2016) and canopy-level transpiration (Irmak et al., 2008; Ding et al., 2014; Wehr et al., 2017; Wehr and Saleska, 2021; Wu et al., 2022).

Moreover, in comparison to the method introduced by Lhomme et al., 2012, the model’s simplicity eliminates the need for intricate techniques to monitor vine transpiration. Instead, it replaces this process with a representative value of the leaf’s stomatal conductance at the canopy surface.

It is noteworthy that the presented model was intentionally designed to address vine water stress, addressing the challenges posed by water and heat stress in Mediterranean climates. The utilization of drip irrigation, implemented in a deficit strategy, aims to optimize water usage and enhance berry quality, while meticulously controlling water stress levels. Although the primary focus remains on water-stressed conditions, it is crucial to emphasize that the model has not undergone testing under non-water-stressed conditions.

## 4 Conclusions

Grapevine leaf stomatal conductance to water vapor is highly influenced by climate and water availability. Under stressful conditions, grapevines tightly regulate canopy transpiration through leaf stomatal conductance.

The developed model for predicting canopy conductance from leaf stomatal conductance (g_sw_), and the meteorological variables net solar radiation and air vapor pressure deficit, scaled by leaf area index, showed good accuracy under stressful conditions.

Several authors have documented a strong correlation between leaf stomatal conductance and soil water content (Dry and Loveys, 1999; Tuzet et al., 2003; Prieto et al., 2010; Costa et al., 2012; Lavoie-Lamoureux et al., 2017), and between soil water content and predawn leaf water potential (Williams and Araujo, 2002; Groenveld et al., 2023). This article emphasizes the role of grapevine water status, in controlling leaf stomatal conductance, and demonstrates a strong correlation between canopy conductance and vine transpiration. The results reveal that, in addition to leaf stomatal conductance, net solar radiation, and air vapor pressure deficit, significantly influence canopy conductance and vine transpiration.

Model validation addressed on a dataset built throughout three data-acquisition campaigns carried out in a commercial vineyard, demonstrated that the proposed biophysical model was an effective predictor of canopy conductance and, consequently, of vine transpiration, under the conditions of this study, as it was previously reported by Lu et al. (2003).

One notable advantage of the presented model, compared to the Penman-Monteith method, is its enhanced simplicity. Importantly, the presented model eliminates the need for intricate methods to monitor vine transpiration and replaces them with a representative value of stomatal conductance of the leaves at the surface of the canopy.

According to the results, monitoring canopy conductance with the presented and simpler biophysical model, can provide valuable information on plant transpiration for efficient vineyard irrigation management in stressful environments. Future work, will rely in validating the model under less stressful environments and on different grape varieties.

## Data availability statement

The raw data supporting the conclusions of this article will be made available by the authors, without undue reservation.

## Author contributions

RE: Conceptualization, Formal Analysis, Investigation, Methodology, Validation, Writing – original draft, Writing – review & editing. AA: Supervision, Validation, Visualization, Writing – review & editing. JA: Supervision, Writing – review & editing.

## Glossary

**Table:**

BL	Big-leaf model
E_c_	Vine canopy transpiration
ET_c_	Crop evapotranspiration
ET_c act_	Actual crop evapotranspiration
g_c_	Canopy conductance to water vapor
g_c est_	Canopy conductance to water vapor estimated by the proposed biophysical model
g_c obs_	Observed/Reference field measured canopy conductance to water vapor
g_s_	Bulk surface conductance
g_sw_	Leaf stomatal conductance to water vapor
LAI	Leaf area index
RH	Relative humidity
R_n_	Net solar radiation
SWC	Soil water content
T_air_	Air temperature
T_min_	Minimum air temperature
T_max_	Maximum air temperature
*U*	Wind speed
VPD	Vapor pressure deficit
Ψ_PD_	Pre-dawn leaf water potential
C_p_	Dry air specific heat at constant pressure
d	Zero-plane displacement height
E_c_	Vine canopy transpiration
e_a_	Actual Vapor pressure
e_s_	Saturation Vapor pressure
e_0_(T_air_)	Saturation Vapor pressure as function of temperature
g_a_	Aerodynamic conductance
h	Canopy height
k	Von Karman’s constant
K_t_	Time unit conversion factor
P	Atmospheric pressure
R_n_	Net solar radiation
R	Specific gas constant
VPD	Vapor pressure deficit
z_m_	Wind speed and Humidity measurement height
z_om_	Roughness length governing transfer of momentum
z_ov_	Roughness length governing transfer of water vapor
Δ	Vapor pressure curve’s slope
ε	Ratio of molecular weight of water Vapor/dry air
λ	Latent heat of water vaporization
γ	Psychrometric constant
ρ	Air density
d	Cohen’s measure for effect size
|E|	Relative error
$g_{c\;est}^{i}$	The *i*-th g_c_ value estimated by the proposed model
$g_{c\;obs}^{i}$	The *i*-th observed g_c_ value given by the Penman-Monteith equation
$\bar{g_{c\;obs}}$	Mean value of the g_c obs_ measures
$\bar{g_{c\;est}}$	Mean value of the g_c est_ measures
MAE	Mean absolute error
N_obs_	Number of samples of the g_c obs_ dataset
N_est_	Number of samples of the g_c est_ dataset
RMSE	Root mean squared error
$s_{obs}^{2}\;$	Variance of the g_c obs_ dataset
$s_{est}^{2}$	Variance of the g_c est_ dataset
*s_p_*	Pooled standard deviation
